# Understanding the complex network of anxiety, depression, sleep problems, and smartphone addiction among college art students using network analysis

**DOI:** 10.3389/fpsyt.2025.1533757

**Published:** 2025-03-04

**Authors:** Jincheng Luo, Jinni Xu, Yifei Lin, Qingquan Chen

**Affiliations:** ^1^ Xiamen Academy of Arts and Design, Fuzhou University, Xiamen, Fujian, China; ^2^ Fujian Medical University, Fuzhou, Fujian, China

**Keywords:** anxiety, depression, sleep problems, smartphone addiction, network analysis

## Abstract

**Background:**

This study employs a network analysis approach to explore the interconnections between anxiety, depression, and sleep problems and smartphone addiction among college students using network analysis, offering a new perspective on these prevalent mental health issues.

**Methods:**

A cross-sectional study was conducted among art students at a public university in the province of Fujian, China. Data were collected using the Generalized Anxiety Disorder Scale-7, Patient Health Questionnaire-9, Pittsburgh Sleep Quality Index, and Mobile Phone Addiction Index. The R package was used in the analysis for statistical analysis, and information was collected using multi-stage sampling as well as stratified sampling. Network analysis was utilized to identify bivariate associations between symptoms, core components, co-occurring patterns, and key nodes within the network. Network stability and accuracy were assessed using the bootstrap method, and network comparisons were conducted across subgroups based on gender, residential condition, and sibling status.

**Results:**

The study included 2,057 participants. The network analysis revealed uncontrollable worry as the most central symptom, with low energy and excessive worry also identified as key symptoms within the network. Bridge symptoms such as daytime dysfunction, self-harm or suicidal ideation, abnormal behavior and speech, and sensory fear were found to be critical in linking anxiety, depression, and sleep problems. The network of comorbid symptoms and smartphone addiction highlighted inefficiency and loss of control as central factors influencing mental health. No significant differences in network characteristics were found across the subgroups, suggesting the universality of the identified network structure.

**Conclusion:**

This study delineates the intricate network of anxiety, depression, sleep problems, and smartphone addiction among college students, identifying key symptomatic intersections and their implications for mental health.

## 1 Introduction

Mental health issues among college students are prevalent worldwide and have long been a concern (1, 2). According to the Diagnostic and Statistical Manual of Mental Disorders (DSM-V) published by the American Psychiatric Association, mental disorders are classified into 22 categories, including depressive disorders, anxiety disorders, and sleep disorders (3). Statistically, approximately 20% of college students worldwide experience mental health disorders within 12 months (1). Zhou et al. (4) reported that the prevalence of anxiety and depression in China was 55.0% and 56.4%, respectively. Another meta-analysis found that the overall prevalence of depression among Chinese college students was 23.8% (5). These data indicate that psychological disorders with anxiety and depression as the main symptoms are widely prevalent in the current college student population, which seriously affects their life and study. Of particular concern is that sleep disorders are often associated with anxiety and depression symptoms (6). It has been reported that the prevalence of sleep disorders among Chinese college students is 25.7%, with up to 23.6% exhibiting insomnia symptoms (7). There is a bidirectional influence relationship between sleep quality and anxiety and depressive symptoms (8–10). A dynamic study of depressed and anxious patients also showed shorter and longer sleep disturbances in people with depression or anxiety (11). This body of research suggests that symptoms of multiple mental disorders often co-occur in individuals and that there may be an interplay between symptoms. In the global context, the co-morbid symptoms of anxiety, depression and sleep disorders are significant, resulting in a wide range of effects.

According to the World Health Organization, depression is expected to become the leading cause of global disease burden by 2030, accounting for 6.2% of all diseases (12). Similarly, according to the World Economic Forum, depression and anxiety cost the global economy about $1 trillion annually. A recent study shows that the percentage of GDP lost due to sleep deprivation in China is 4.62% (13). The significant economic burden of mental disorders globally emphasizes the urgency of conducting in-depth research on mental health issues. Mental disorders are recognized as significant risk factors for suicidal behavior. Depressed individuals represent the majority of suicide-related deaths (14), and over 70% of those with prior suicidal behavior meet the diagnostic criteria for anxiety disorders (15). Additionally, sleep disorders are critical risk factors for both suicidal ideation and behavior (16). A meta-analysis of Chinese college students found a moderate association between depressive symptoms and suicidal ideation (17). These conditions, characterized by a growing social burden and poor prognosis, highlight the pressing necessity for comprehensive research intomental disorders, such as anxiety, depression, and sleep disorders.

Recent studies have increasingly recognized the limitations of relying solely on total scores from standardized scales to assess the mental health of college students. Instead, there is a growing emphasis on evaluating individual symptoms and their interrelationships. The network theory of mental disorders, proposed by Borsboom, offers a novel framework for understanding these conditions, suggesting that mental disorders arise from the interactions between symptoms (18). This theory employs a network analysis approach, which conceptualizes symptoms as a network of interrelated nodes, with the connections between these nodes representing causal relationships (19). Symptoms with high centrality within the network may act as core symptoms, exerting the strongest influence on other symptoms. Intervening on these central symptoms may disrupt the network of interactions, potentially leading to improvements in peripheral symptoms. Numerous studies in the field of mental disorders have demonstrated the effectiveness and applicability of this approach (20, 21).

Smartphones, as the most prevalent communication devices, have seen their addiction among college students escalate into a grave concern amidst the relentless progress of mobile technology (22). A review and analysis by Ratan et al. (23) highlighted a significant association between smartphone addiction and mental health problems. Several studies have demonstrated that smartphone addiction is a risk factor for depression and sleep disorders. A meta-analysis specifically focused on college students also provided strong evidence that smartphone addiction is positively correlated with anxiety, depression, and sleep problems (24–26). Among college students, the reported prevalence of problematic smartphone use and smartphone dependence was 78.3% and 7.4%, respectively, indicating that a substantial number of students remain at risk for mental health issues (27). Therefore, it is crucial to conduct a comprehensive investigation into the relationship between smartphone addiction and mental health problems such as depression, anxiety, and sleep disorders to enhance the overall well-being of college students. It is academically and practically important to study the association between cell phone addiction and mental health problems, especially the co-morbidity of anxiety, depression, and sleep disorders.

More recently, researchers have proposed incorporating symptom measures related to external events. They argue that this approach should not be limited to psychiatric symptoms alone but should also consider a broader range of theoretically relevant factors, including risk and protective factors. Such an approach could offer a deeper understanding of the complex mechanisms that either promote or prevent the development of mental disorders (28). Risk factors, in particular, provide valuable insights into the underlying causes of mental illness. For instance, a meta-analysis of 66 studies found that experiences of sexual harassment were significant predictors of depressive outcomes, highlighting the critical role of childhood experiences in increasing the risk of depression (29). By integrating symptoms of risky smartphone addiction with anxiety, depression, and sleep problems, the network model offers a more comprehensive framework for examining the relationship between smartphone addiction and the development of mental health disorders. Furthermore, considering factors such as gender, living conditions, and family cohort status can provide valuable insights into these dynamics. Previous studies have predominantly employed the Network Comparison Test (NCT), a resampling-based alignment test, to evaluate the overall strength and marginal connections between subgroups. This approach offers an effective method for analyzing differences in network characteristics across various groups (28).

The challenges posed by mental health problems are complex. The key issues addressed in this study are the prevalence of mental health problems among college students and the impact that external factors and risk factors have on them. At the same time, the overarching goals of our study are (a) to explore the symptoms that play an activating, maintaining, and connecting role in the psychopathology network with respect to anxiety, depression, and sleep problems. Psychopathology research is constantly evolving, so the second goal we considered was (b) exploring how co-morbid symptoms and risky smartphone addiction manifestations are interconnected, discovering core mental health deteriorating behaviors, and investigating whether these associations vary by gender, education level, familial siblings, and mental health status. These studies may be able to provide additional new ideas in college student mental health and move one step closer to more precise health promotion as well as non-pharmacological intervention strategies.

## 2 Methods

### 2.1 Study design

We conducted a cross-sectional study in a comprehensive national key university that admits new students nationwide every year, thus ensuring a representative sample. Data collection was carried out with the Generalized Anxiety Disorder Scale-7, Patient Health Questionnaire-9, Pittsburgh Sleep Quality Index, and Mobile Phone Addiction Index. Network analysis was then conducted to identify bivariate associations between symptoms, core components, co-occurring patterns, and key nodes within the network. Finally, network stability and accuracy were assessed, and network comparisons were conducted.

### 2.2 Participants

This study was conducted among art students. A multi-stage sampling method was used, with stratified sampling throughout, followed by a randomly selected survey. Similar to relevant studies (30, 31), the WeChat-integrated “Questionnaire Star” program was used to collect data. Electronic questionnaires were distributed to university students through WeChat and Tencent QQ platforms.

Measures taken to ensure data quality included limiting submissions to one per IP address, excluding responses with completion times outside the 6- to 10-minute range, eliminating incomplete questionnaires, and discarding questionnaires with contradictory answers.

Efficacy analysis is often used to determine the sample size required for a study. Research by Epskamp and Fried suggests (32) that simulating data in a given network model and expected network structure using the netSimulator function enables the exploration of appropriate sample sizes required to detect true effect sizes. Due to the node richness of the network, we would need a larger sample size to achieve the same level of reproducibility. Therefore, we simulated data for a network model that included both co-morbid symptoms and cell phone addiction networks, and the results are shown in **Supplementary Figure S1**.

Eventually, a total of 2,057 eligible participants were included. Prior to participation, all subjects provided electronic informed consent.

### 2.3 Measurements

#### 2.3.1 Generalized Anxiety Disorder Scale-7

We used the Generalized Anxiety Disorder Scale-7 questionnaire to assess the level of anxiety disorders in the population (33). Its validity as a screening tool for anxiety in the Chinese population has been established (34). The scale consists of seven items based on the Diagnostic and Statistical Manual of Mental Disorders, Fourth Edition (DSM-IV) criteria for generalized anxiety disorder (35). Each item is scored on a 4-point scale, ranging from 0 (not at all) to 3 (almost every day). The total score ranges from 0 to 21, with higher scores indicating more severe anxiety symptoms. Specifically, a GAD-7 score of ≥ 10 is used to identify generalized anxiety disorder (36).

#### 2.3.2 Patient Health Questionnaire-9

We used the Patient Health Questionnaire-9 to assess depressive symptoms in the population, which is considered one of the most reliable depression screening tools (37, 38). Based on the DSM-IV, the PHQ-9 consists of nine questions, with each question scored from 0 to 3, yielding a total score of up to 27 points. According to the PHQ-9 scoring scale, a score below 5 indicates minimal or no depressive symptoms, while a score above 5 suggests an elevated risk for depression (39, 40). Moreover, the PHQ-9 has been extensively validated in the Chinese population (41, 42).

#### 2.3.3 Pittsburgh Sleep Quality Index

The Pittsburgh Sleep Quality Index is a validated instrument for assessing sleep quality, encompassing seven dimensions of sleep characteristics: subjective sleep quality, sleep latency, sleep duration, sleep efficiency, sleep problems, use of sleep medications, and daytime dysfunction (43). The PSQI is a self-reported questionnaire consisting of 19 items (44). In the Chinese population, a total PSQI score greater than 7 indicates poor sleep quality, with a sensitivity of 98.3% and a specificity of 90.2% (28).

#### 2.3.4 Mobile Phone Addiction Index

The Mobile Phone Addiction Index is a straightforward tool primarily designed to assess mobile phone addiction in adolescents and college students (45). Based on the DSM-IV, the MPAI consists of 17 questions that primarily address four dimensions of mobile phone use: loss of control, withdrawal, avoidance, and inefficacy. The MPAI uses a 5-point Likert scale, where a score of 1 to 5 reflects increasing levels of addiction, with higher scores indicating greater mobile phone dependence. The Chinese version of the scale has been extensively used among Chinese students, demonstrating high reliability and excellent validity (45).

### 2.4 Statistical analysis

First, we employed descriptive statistics to summarize the item scores for each scale, as well as demographic information about the population. Second, we constructed comorbidity networks for anxiety, depression, and sleep problems, in addition to a combined network for comorbid symptoms and smartphone addiction. Network analysis reveals the structure and characteristics of complex mental disorder systems that cannot be fully explained by traditional regression or latent variable analyses (46). Before constructing the network, potential item redundancy was assessed using the goldbricker function from the R package network tools (version 1.5.1). Following Jones’ guidelines, items were considered redundant if the proportion of significantly distinct central correlations between two variables and other items was below 25%. All statistical analyses were performed using R Version 4.3.2.

### 2.5 Network analysis

#### 2.5.1 Network estimation

The Pairwise Markov Random Field (PMRF) is commonly employed in cross-sectional studies. In our analysis, we applied the Gaussian Graphical Model (GGM) to estimate a network of partial correlation coefficients (47). This partial correlation network, based on weighted correlation networks, evaluates the relationship between two nodes while controlling for all other variables in the network. To reduce spurious connections and enhance the clarity of the network, we applied the graphical least absolute shrinkage and selection operator (LASSO) for regularization (48). The Extended Bayesian Information Criterion (EBIC) was used to select the optimal model fit, with a default tuning parameter of 0.5. For network layout, we employed the Fruchterman-Reingold algorithm, which positions nodes with stronger correlations closer together. Network estimation and visualization were performed using the R packages graph (version 1.9.8) and bootnet (version 1.5.6) (49). In the resulting graphs, items are represented as nodes, and the edges between them vary in thickness to indicate the strength of associations, with blue representing positive correlations and red indicating negative correlations.

To quantify the significance of individual nodes within a network, centrality metrics are computed, reflecting the likelihood that the activation of one node influences others (50). Key centrality measures include betweenness, closeness, and strength. However, previous studies have indicated that betweenness and closeness may not reliably capture node importance (51). Therefore, this study primarily focuses on strength as the centrality metric, which quantifies the total absolute edge weights between a node and its directly connected neighbors, emphasizing the strength of direct connections without intermediaries. Nodes with higher strength are considered more central to the network. In addition, bridge centrality was assessed using the bridge function from the R package network (version 1.5.1) to identify nodes that serve as connectors between distinct communities within the network (52). Bridge strength measures the total edge weight between a community node and all nodes outside its community, highlighting the importance of these nodes in linking disparate parts of the network. Finally, the R package MGM (version 1.2–14) was employed to compute predictability, which indicates the extent to which the variation in a node can be explained by its connected neighbors (53). In the network visualization, the area surrounding each node’s cycle represents its predictability value.

#### 2.5.2 Network stability and accuracy

We assessed network stability and accuracy using the bootstrap method from the R package bootnet (version 1.5.6), employing three key processes. First, we applied a case-dropping bootstrap procedure to evaluate the stability of strength and bridge strength, systematically removing portions of the data without substantially altering the network structure. Stability was quantified using the Correlation Stability Coefficient (CS-C), which reflects the maximum proportion of the sample that can be omitted without significantly affecting the network. A CS-C value of 0.25 or higher is considered acceptable, with values exceeding 0.5 being particularly desirable. Next, we evaluated the accuracy of edge weights by applying a non-parametric bootstrap method to calculate confidence intervals (CIs) (54). Narrower intervals indicate more precise and reliable estimates. Finally, we compared differences between edges and nodes by conducting bootstrapped paired differences in both edge weights and centrality measures.

#### 2.5.3 Network comparison

Finally, to explore potential differences in online characteristics related to co-morbidities and cell phone addiction among college students, we analyzed the data based on gender, residential condition, and sibling status. Gender was categorized as “Male” and “Female,” residential condition as “Rural” and “Urban,” and sibling status as “Only child” and “Non-only child.” A permutation test with 1,000 iterations was performed using the Network Comparison Test package (version 2.2.2) in R to assess statistical differences in global strength (the absolute sum of all edge weights) and network structure (the distributions of edge weights) across the subgroups (55). Subsequently, we applied the Bonferroni-Holm correction to adjust for multiple comparisons and evaluate differences in strength at the individual edge level between the networks.

## 3 Results

### 3.1 Characteristics of the study sample

**Table 1** presents the demographic characteristics of the participants in this study. A total of 2,057 participants were included, with a mean age of 20.22 years (SD = 1.52). In terms of mental health, 1,102 participants (53.57%) reported symptoms of anxiety (GAD-7 total score ≥ 5), 1,260 participants (61.25%) reported symptoms of depression (PHQ-9 score ≥ 5), 1,217 participants (59.16%) reported sleep problems (PSQI total score ≥ 8), and 1,479 participants (71.90%) reported cell phone addiction (additional details are shown in **Supplementary Table S1**).

**Table 1:** Demographic characteristics of the study participants (n = 2,057).

Variables	Mean/N	SD/%
Age (year)	20.22	1.52
Anxiety (GAD-7)	5.69	5.46
Depression (PHQ-9)	7.29	6.37
Pittsburgh sleep quality index (PSQI)	8.38	3.12
Mobile phone addiction index (MPAI)	43.71	14.15
Gender		
Male	645	31.36%
Female	1412	68.64%
BMI (kg/m2)		
Underweight (≦ 18.4)	450	21.88%
Normal weight (18.5–23.9)	1406	68.35%
Overweight (≧ 24)	201	9.77%
Residence		
Urban	1090	52.99%
Rural	967	47.01%
Family sibling status		
Only child	764	37.14%
Non-only child	1293	62.86%
Coffee		
Rarely or never	855	41.57%
Occasionally	947	46.04%
Often or almost every day	255	12.40%
Tea		
Rarely or never	668	32.47%
Occasionally	1112	54.06%
Often or almost every day	277	13.47%
Smoke		
Rarely or never	1849	89.89%
Occasionally	110	5.35%
Often or almost every day	98	4.76%
Alcohol		
Rarely or never	1531	74.43%
Occasionally	483	23.48%
Often or almost every day	43	2.09%
Anxiety (GAD-7)		
No anxiety (0–4)	955	46.43%
With anxiety (5–21)	1102	53.57%
Depression (PHQ-9)		
No depression (0–4)	797	38.75%
With depression (5–27)	1260	61.25%
Pittsburgh sleep quality index (PSQI)		
Normal sleep (0–7)	840	40.84%
Have sleep problems (8–21)	1217	59.16%
Mobile phone addiction index (MPAI)		
No addiction (0–33)	578	28.10%
Mobile phone addiction (34–85)	1479	71.90%

### 3.2 Anxiety, depression and sleep problems network

**Figure 1:**
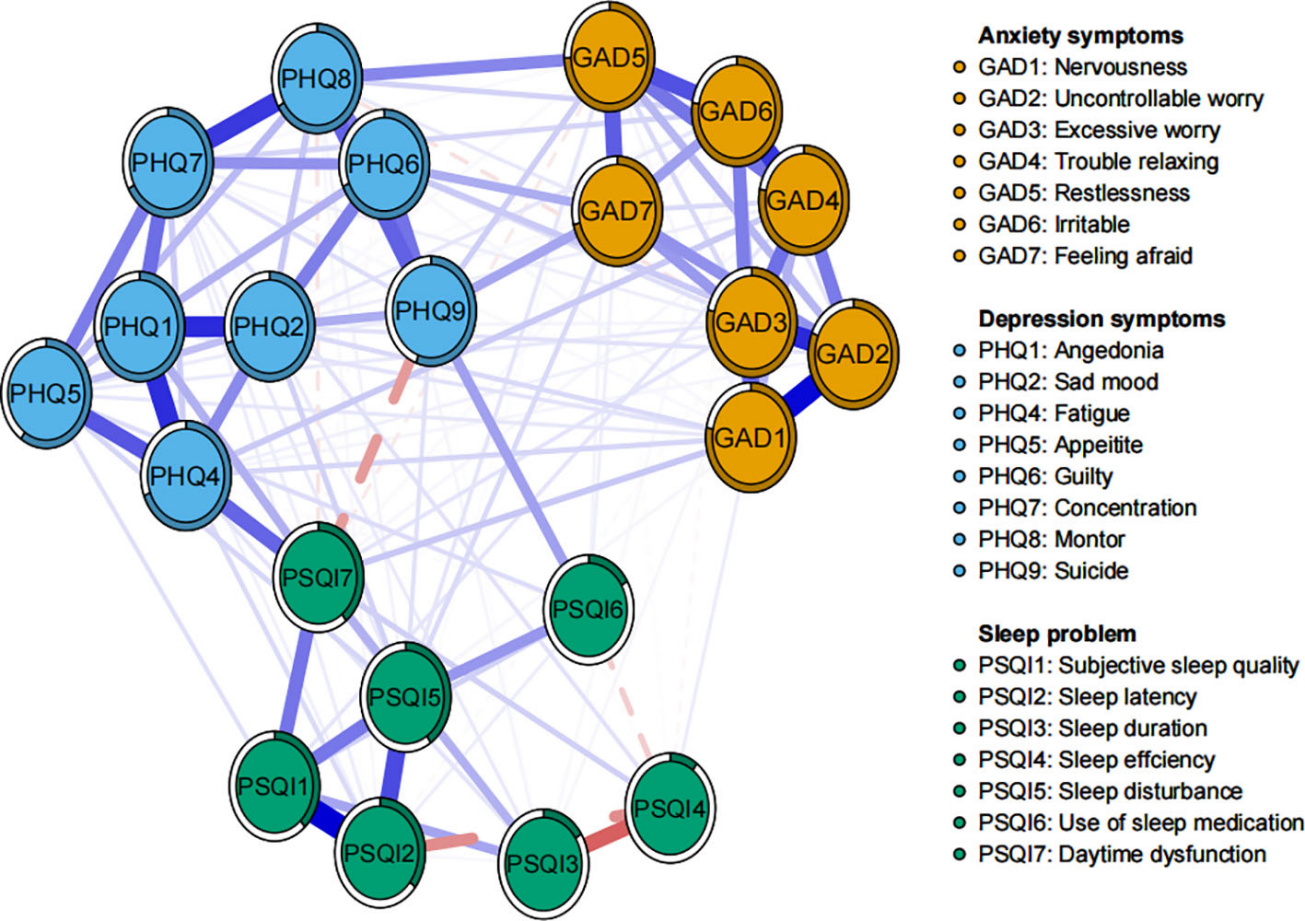
Network structure of anxiety, depression and sleep problems among study participants.

As shown in **Figure 1**, the network structure, which includes anxiety, depression, and sleep problems, contained 137 non-zero edges out of a possible 231, resulting in a network density of 0.59 and an average edge weight of 0.039. The two most prominent edges were within specific communities: subjective sleep quality and sleep latency (PSQI1-PSQI2), and nervousness and uncontrollable worry (GAD1-GAD2) (**Supplementary Table S2**). Key connections between different communities included: self-harm or even suicide and use of sleep medication (PHQ9-PSQI6), low energy and daytime dysfunction (PHQ4-PSQI7), restlessness and abnormal behavior or speech (GAD5-PHQ8), and feeling afraid and self-harm or even suicide (GAD7-PHQ9) (**Supplementary Table S2**). Furthermore, uncontrollable worry and excessive worry exhibited the highest predictability values (0.815 and 0.787, respectively), while the lowest predictability (0.173) was observed for sleep efficiency (PSQI4). The overall average predictability was 0.574, indicating that, on average, more than half of the variance in the nodes could be explained by their neighboring nodes (**Supplementary Table S1**). Network stability and accuracy tests, presented in **Supplementary Figure S4**, showed that both strength and bridge strength centrality values were 0.75, surpassing the recommended threshold of 0.5.

**Figure 2:**
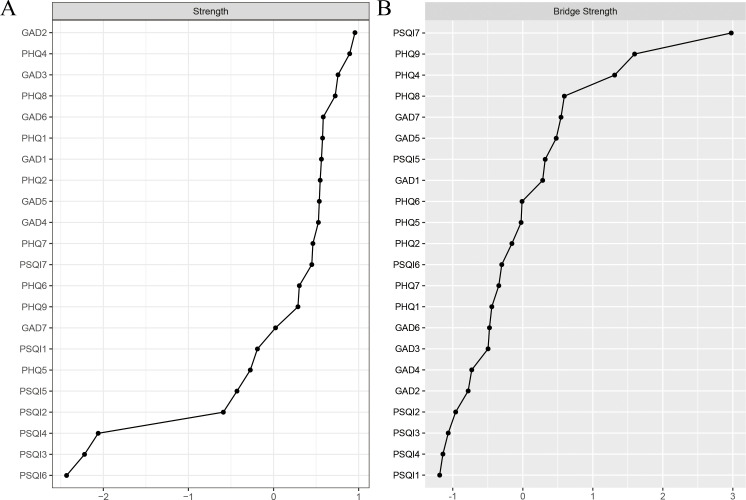
Standardized strength and bridge strength centrality of network structure of anxiety, depression and sleep problems among study participants (z-scores). **(A)** strength centrality; **(B)** bridge strength centrality.

The centrality plot (**Figure 2A**) revealed that GAD2 exhibited the strongest centrality, followed by PHQ4 and GAD3, suggesting that these nodes occupy the most central and influential positions within the network. According to Jones (50), bridge symptoms were identified using the 80th percentile threshold for bridge centrality. As shown in **Figure 2B**, PSQI7, PHQ9, PHQ4, PHQ8, and GAD7 exhibited the highest bridge strengths, highlighting them as key bridging symptoms that connect comorbid anxiety, depression, and sleep problems. Bootstrap difference tests for node strength and bridge strength (**Supplementary Figure S4**) further confirmed that these nodes were statistically significantly stronger than other nodes in the network.

### 3.3 Co-morbid symptoms and smartphone addiction networks

**Figure 3:**
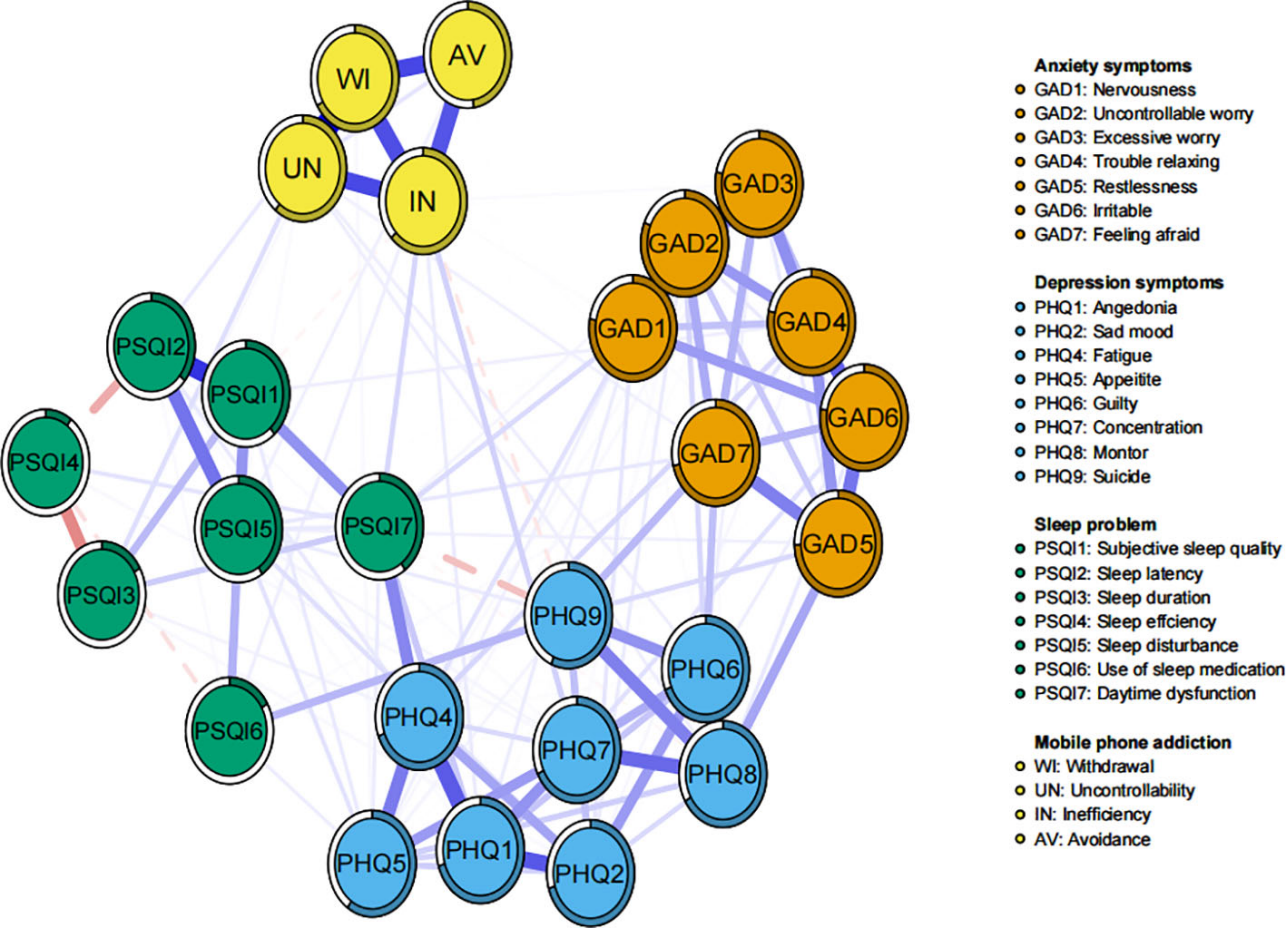
Network structure of comorbid symptoms and mobile phone addiction among study participants.

The network of comorbid symptoms and smartphone addiction is depicted in **Figure 3**, with stability and accuracy tests provided in **Supplementary Figure S5**. In terms of power centralization, uncontrollable worry and low energy emerged as the most significant and influential comorbid symptoms. Meanwhile, withdrawal and inefficiency were identified as the top two central MAPIs (**Supplementary Figure S2**).

In terms of correlations, Inefficiency and Uncontrollability exhibited the strongest negative associations with co-morbid symptoms. Furthermore, by examining the individual connections between each specific smartphone addiction node and the broader co-morbid symptom communities, Inefficiency demonstrated the most significant bridging role across the anxiety, depression, and sleep problem communities (**Supplementary Table S3**; **Supplementary Figure S3**). In contrast, Inefficiency and Uncontrollability were predominantly positively associated with other co-morbid symptoms. These findings suggest that Inefficiency and Uncontrollability are core behaviors that significantly impact mental health.

### 3.4 Comparison based on gender, place of residence, family siblings and mental health status

Network comparison tests revealed invariance in overall strength and network structure across the three groups (**Supplementary Figures S6**-**S8**). In terms of overall strength, no significant differences were found between the subsamples based on mental health status (male: 12.76 vs. female: 12.21, p = 0.102; urban: 12.12 vs. rural: 11.98, p = 0.590; only child: 11.95 vs. non-only child: 12.24, p = 0.340). Regarding the comparison of network structure, no significant differences were observed across the three subsamples (gender: M = 0.17, p = 0.199; residence: M = 0.16, p = 0.220; family sibling status: M = 0.19, p = 0.054). Furthermore, after applying the Bonferroni-Holm correction, all edge weights across the three subsamples remained non-significant (p > 0.05).

## 4 Discussion

There is a clear correlation between anxiety, depression and sleep problems. In a survey of mood states, the majority of pre-service teachers were found to be depressed and anxious (56), again suggesting some correlation between the two. In this study, we assessed both core and bridging symptoms within a comorbid network of anxiety, depression, and sleep problems, identifying key behaviors that influence mental health. Our survey of art students at a public university in the province of Fujian, China revealed that more than half reported symptoms of anxiety or depression, while between one-third and two-thirds experienced sleep-related issues. Focusing on sleep, the relationship between sleep problems and both anxiety and depression highlights the complex nature of mental health disorders. The Cell Phone Addiction Index includes four factors: loss of control, withdrawal, avoidance, and inefficacy. By employing a comprehensive, multi-dimensional network approach, we can analyze the intricate associations between cell phone addiction and specific mental health conditions. The following section will discuss the results of these network analyses.

### 4.1 Network results for anxiety, depression and sleep problems

Uncontrollable worry exhibited the highest centrality in terms of intensity, highlighting its critical role in activating and sustaining the comorbid network of depression, anxiety, and sleep problems. This phenomenon has been widely observed in older populations as well (57). Uncontrollable worry is a hallmark of generalized anxiety disorder and has been identified as a potential target for interventions aimed at treating both anxiety and depression (58, 59). Li W et al. (60) found uncontrollable worry to be the most central symptom in network analyses of anxiety and depression among adolescents and young adults with cancer. Similarly, in a network analysis of depression and anxiety symptoms in college students during the later stages of the COVID-19 pandemic, uncontrollable worry was also confirmed as a central symptom (61). In different studies, “uncontrollable worry” has been confirmed to be strongly associated with anxiety and depression, which is consistent with our findings. The innovative inclusion of the sleep problem community in the network analysis of our study further illustrates the consistent impact of uncontrollable worry on depression, anxiety, and sleep problems, and provides a more comprehensive picture of the role of “uncontrollable worry” in mental health-related domains. Additionally, low energy and excessive worry emerged as the second most common symptoms. Among these, low energy demonstrated a strong bridging centrality and can be classified as a bridging symptom. Low energy, a core feature of depression, manifest primarily as fatigue or exhaustion (62). An epidemiological study in France found that 41.2% of patients reported persistent fatigue symptoms (63). Furthermore, research has shown that mental illness and somatic complaints may contribute to the development of fatigue symptoms (64), suggesting that somatization resulting from the long-term effects of mental health disorders may manifest as a state of low energy. Network analysis by Zhao N et al. (65) confirmed that low energy serves as a bridging symptom linking depression and insomnia communities, a finding that supports our study. Moreover, a cross-sectional study analyzing a network model of comorbid anxiety and insomnia symptoms in the presence of depressive symptoms identified low energy as one of the bridging symptoms, further reinforcing the validity of our results (66).

Nodal bridging intensity centrality may offer valuable insights into identifying key bridging symptoms within the connectivity and development of mental disorders. Beyond low energy, several other bridging symptoms emerged in the current network, including daytime dysfunction, self-harm and even suicide, abnormal behavior and speech, and sensory fear. Daytime dysfunction, a prominent manifestation of sleep problems (67), has previously been identified as a bridge symptom in networks linking childhood trauma, sleep disorders, and depression (68). A study by Sun C et al. (28) on anxiety, depression, sleep problems, and the promotion of healthy lifestyles among Chinese college students reported findings consistent with our own, providing strong support for our study’s conclusions. Suicidal ideation is a significant concern among college students worldwide (2). As a psychological issue with a high global prevalence, self-harm and suicide have been recognized as critical intervention targets for adolescent mental health (69). Numerous studies have explored the association between mental health and suicidal ideation and behavior. For example, research indicates that anxiety symptoms are present in over 70% of individuals who attempt suicide (15), and depression is a triggering factor for suicide in more than half of suicide decedents and 20%-48% of suicide attempters (70). Our study reveals that abnormal behavior and speech is another key bridge symptom, strongly associated with suicidal behavior. Previous research has noted that abnormal behavior and speech serve as a bridge between depression and sleep disorders (65). Furthermore, a web-based study conducted during the pre-and mid-pandemic phases of COVID-19 confirmed that abnormal behavior and speech important bridge symptoms connecting anxiety, depression, and sleep problems, supporting our findings (71). In addition, our study identified sensory fear as another critical bridge symptom, with a strong association with suicidal ideation. Epidemiological studies have shown that 75% of lifetime psychiatric disorders manifest before the age of 24, with anxiety disorders typically emerging in early adolescence or young adulthood (72). Factors such as student debt, academic stress, and family separation, which are particularly relevant to college students, increase the likelihood of suicidal ideation in individuals with anxiety disorders (73).

### 4.2 Network results for mobile phone addiction

In 2020, the World Health Organization recognized addiction to digital technology as a global health issue. A study reported that the global prevalence of cell phone addiction is 28.3%, Smartphone addiction is significantly associated with mental health problems in adolescents, highlighting the significance of our research. Our findings revealed that “inefficacy” was identified as one of the two most central MAPIs in the network, suggesting it lies at the intersection of mental disorders and cell phone addiction. When quantifying the relationship between specific smartphone addiction factors and changes in comorbid symptoms, inefficacy emerged as the most prominent node. Specifically, inefficacy demonstrated a positive correlation with comorbid symptoms and may contribute to the exacerbation of anxiety, depression, and sleep problems. Sleep disruption and deprivation exert detrimental impacts on brain function, impairing the management of adaptive systems (74). It is noted that chronic maladaptive and inefficient behaviors can create stressful situations, potentially leading to persistent anxiety (75). These findings imply a relationship between inefficacy and symptoms of anxiety, depression, and sleep problems. Nevertheless, our search has not yielded any relevant studies on the impact of inefficacy on these symptoms to date, suggesting that our study may serve as a valuable complement to the existing research.

Our study identifies loss of control as a key behavioral factor influencing mental health. Previous research has linked smartphone addiction to impaired self-regulation (76). Specifically, a study by Chun et al. (77) examined the behavioral and neurological responses to excessive smartphone use, finding that it may disrupt cognitive control over emotional processing. These findings suggest a critical connection between uncontrolled smartphone addiction and mental health issues, thereby providing robust support for our research.

NCT analysis and centrality quantification confirmed the broad applicability of inefficacy and loss of control as key factors influencing mental health. Additionally, no significant differences were observed when comparing the intensity of smartphone addiction behaviors and their impact on mental health symptoms across different subgroups. Previous studies have established an association between depression, anxiety, and smartphone addiction, with some evidence suggesting that this relationship may vary by gender (78, 79). For instance, a longitudinal study by Jun-Qi Ma et al. (80) found that in rural areas, increased smartphone use could have a more pronounced negative effect on adolescents’ mental health. The discrepancy between these findings and our own may be attributed to our use of network analysis, which provides a different methodological approach. Further research is needed to explore these relationships in more depth.

### 4.3 Strengths, limitations and prospects

Our study has several strengths. First, this study targeted Chinese college students and used network analysis to identify core and bridging symptoms of three common mental disorders. Second, it uniquely incorporated cell phone addiction into the network, explored the association of these behaviors with mental disorders, and identified core symptoms that affect mental health behaviors, providing direction for addressing the current pandemic of cell phone addiction. The analysis suggests that cell phone addiction behaviors contribute to the development of mental disorders by primarily influencing core and bridging symptoms. Finally, a web-based comparative test was conducted to further understand the different effects or generalizability of these behaviors in the student population.

Of course, our study has some limitations; first, although the cross-sectional design of the study precludes causal inferences, longitudinal data are needed to elucidate the complex mechanisms of the interactions between symptoms and cell phone addictive behaviors. Second, future studies should incorporate protective factors to gain a comprehensive understanding, as risk factors may exacerbate mental disorders while protective factors may counteract these effects and could provide more valid recommendations. Third, our study was conducted among art students at a comprehensive university, and despite our efforts to minimize bias, the generalizability of the findings to other populations needs to be cautiously explored. Finally, it is important to recognize that all sources of sample information in this study were self-reported, and subjective factors this may have had an impact on the accuracy of the analysis.

Through network analysis, this study examined the central and bridging symptoms of depression, anxiety, and sleep problems in college students, and explored the link between co-morbid symptoms and cell phone addiction, providing a clearer structure for understanding the relationship between these symptoms. These findings provide some clues for improving student mental health interventions in the face of similar disorders in the future.

## 5 Conclusions

The study identifies key symptoms like uncontrollable worry and low energy were central to the mental health network, with bridge symptoms such as daytime dysfunction and suicidal ideation linking comorbid conditions. The integration of MPAI into the network model revealed its association with mental health, emphasizing the need to target withdrawal and inefficiency in interventions. This research provides a basis for developing targeted, non-pharmacological mental health strategies for college students in the arts, highlighting the intersection of mental health disorders and technology use.

## Data Availability

The raw data supporting the conclusions of this article will be made available by the authors, without undue reservation.
