# Supplementary material for: Understanding the complex network of anxiety, depression, sleep problems, and smartphone addiction among college art students using network analysis
Source: Front Psychiatry. 2025 Mar 4;16:1533757. doi: 10.3389/fpsyt.2025.1533757 (PMC11913800; doi:10.3389/fpsyt.2025.1533757)
Supplement: Supplementary file 1 [file SupplementaryFile1.docx]

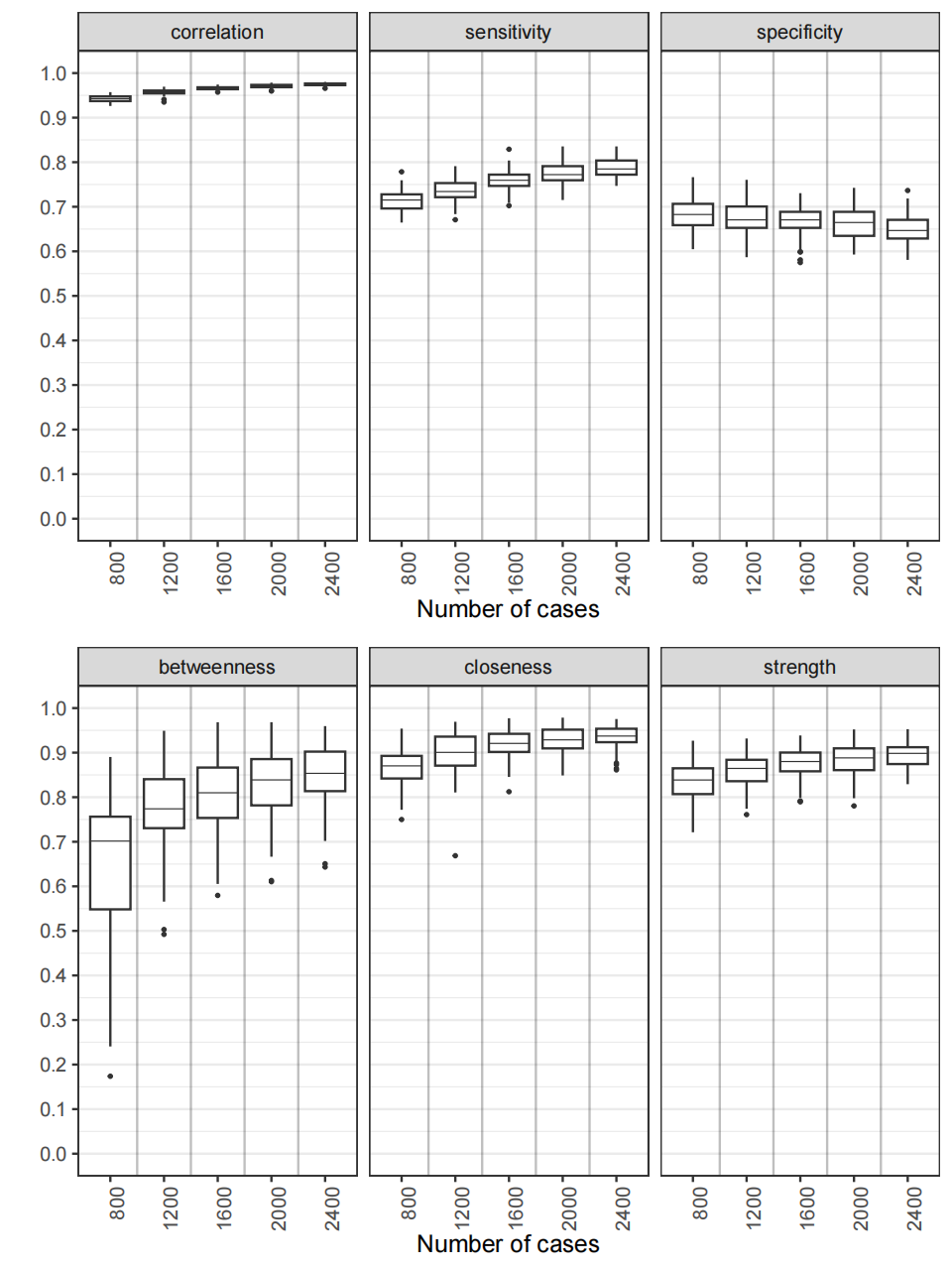


**Figure S1.** The power analysis simulation results of Comorbid symptoms and mobile phone addiction network.


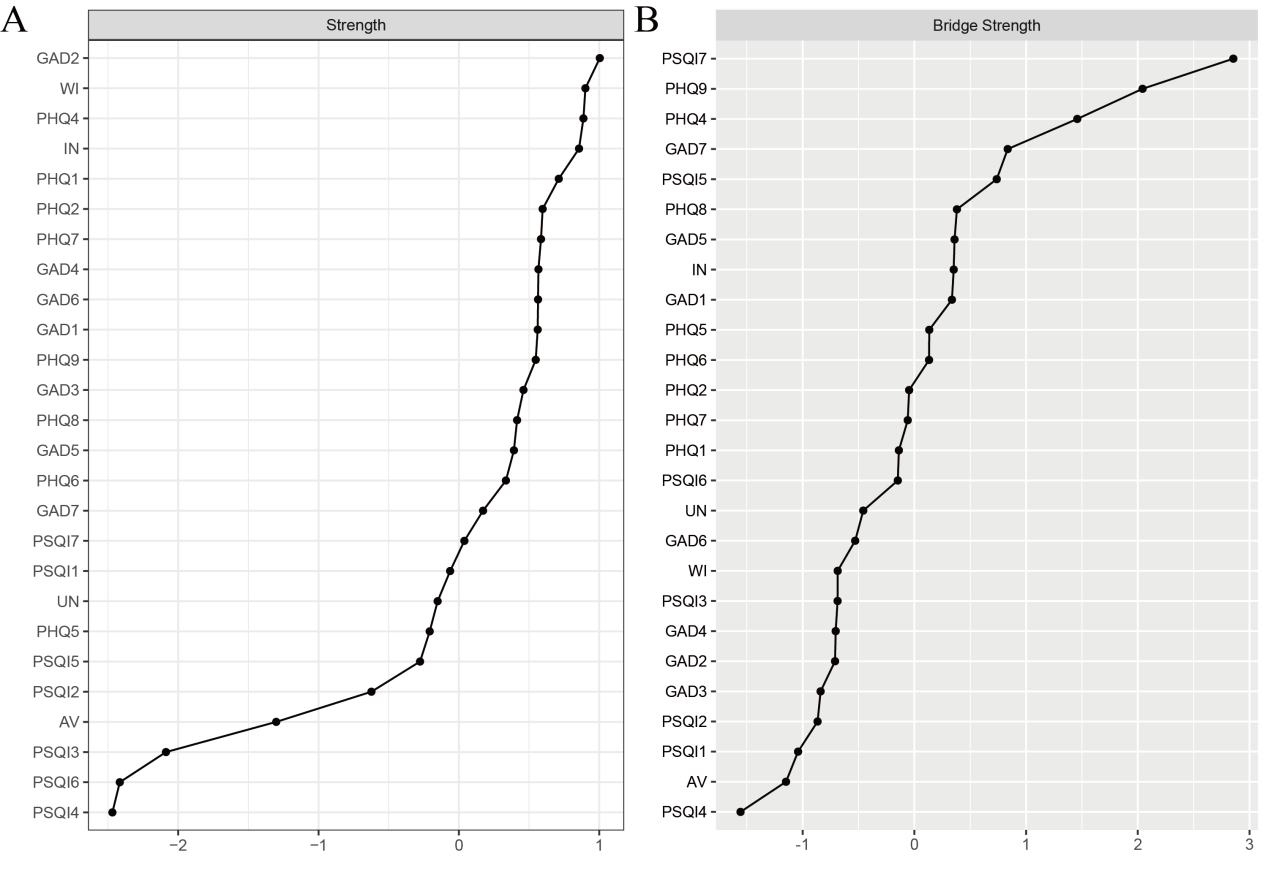


**Figure S2.** Standardized strength and bridge strength centrality of network structure of Comorbid symptoms and mobile phone addiction network among study participants (z-scores). (A) strength centrality; (B) bridge strength centrality.


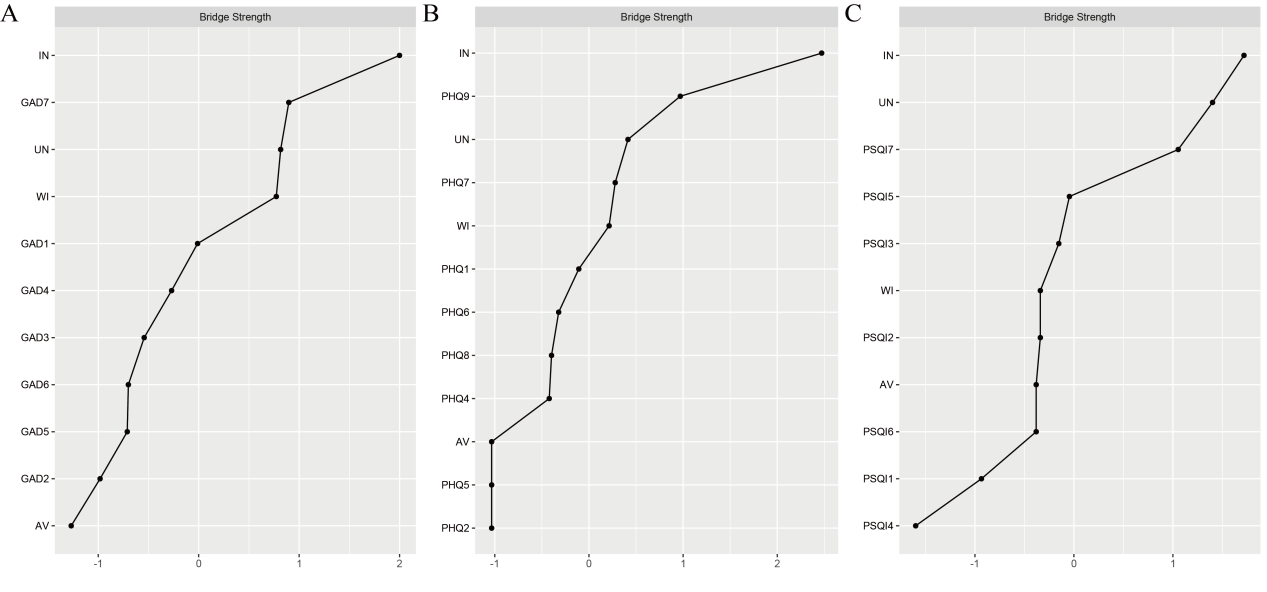


**Figure S3.** Standardized bridge strength centrality plot between mobile phone addiction to Symptoms of Anxiety, Depression, Sleep problems (z-scores).


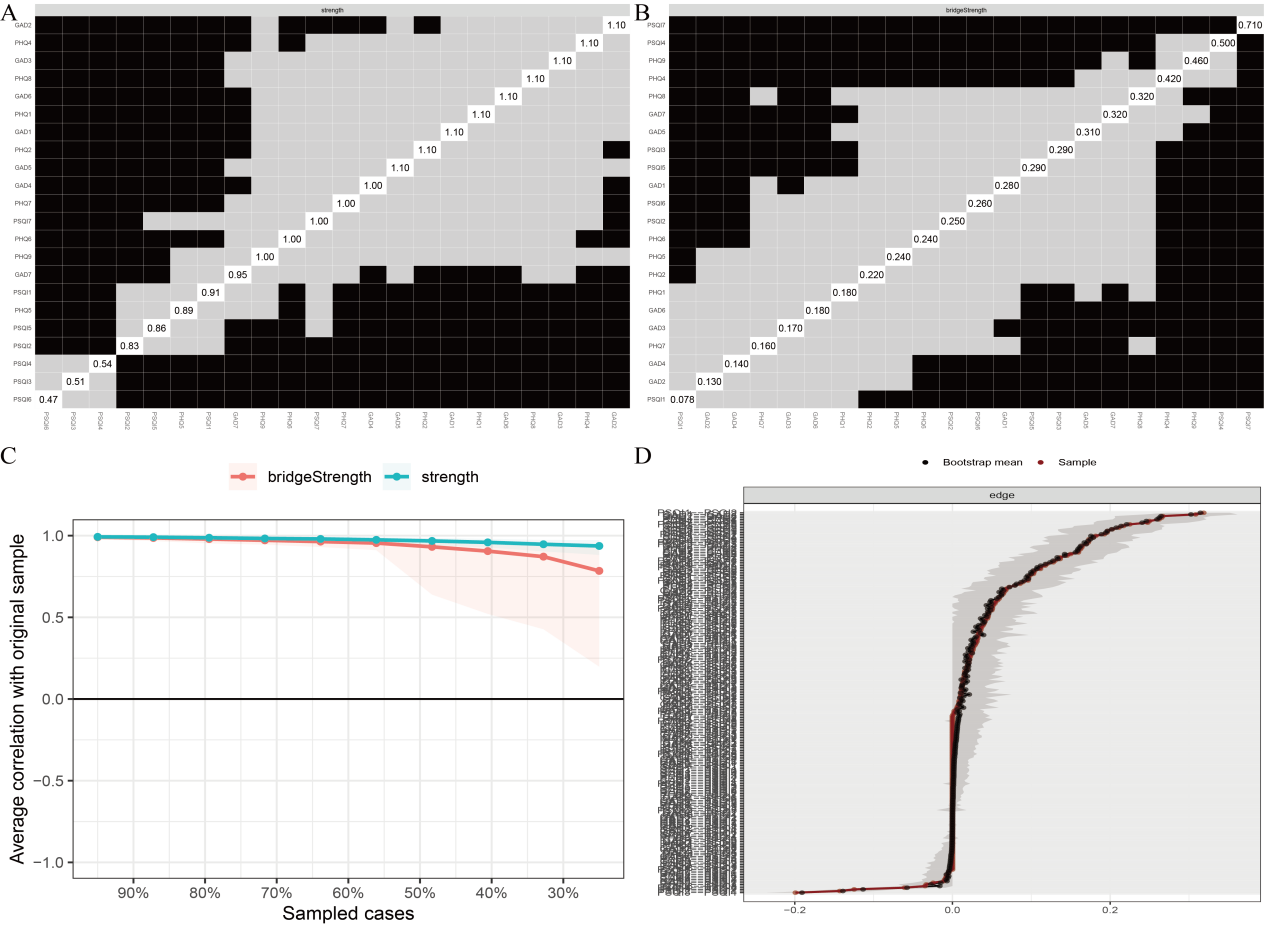
**Figure S4.** Results of Network Stability and Accuracy for Anxiety, Depression, and Sleep Problems. (A) Nonparametric bootstrapped difference test for strength. (B) Nonparametric bootstrapped difference test for bridge strength. Gray boxes indicate that node strength/bridge strength does not show significant differences, while black boxes indicate significant differences. The numbers within the white boxes (i.e., along the diagonal) represent the values of node strength/bridge strength. (C) Post-hoc analysis of the stability of node strength and bridge strength. The x-axis shows the percentage of the original sample used at each step, while the y-axis reflects the average correlations between the centrality metrics in the original network and those from the re-estimated networks after excluding progressively larger percentages of cases. The lines represent the correlations for strength and bridge strength. (D) Bootstrapped 95% confidence intervals for estimated edges. The red line indicates the edge estimated from the sample, and the gray area represents the 95% bootstrapped confidence interval. The x-axis shows the edges, with specific edges identified along the y-axis by the gray lines.


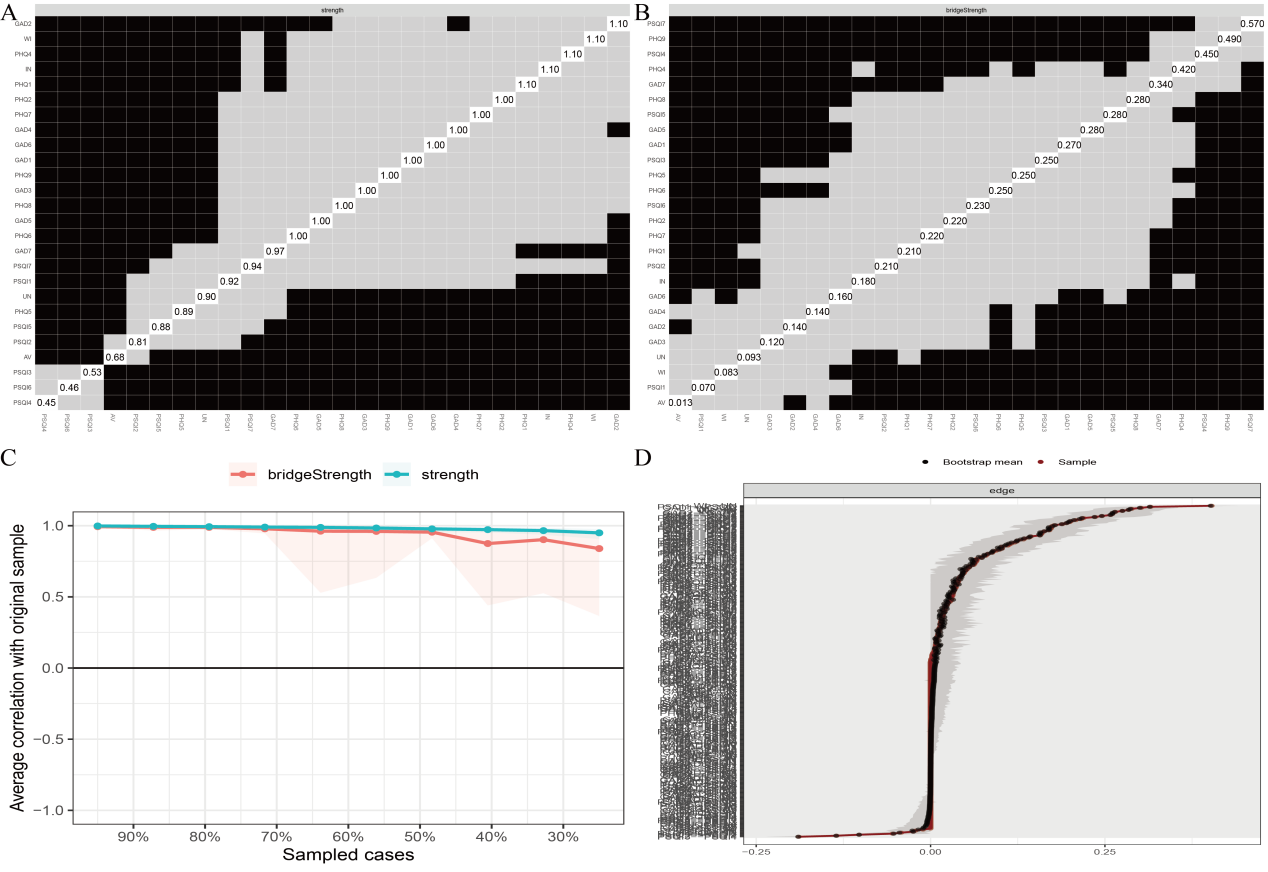


**Figure S5.** Results of Network Stability and Accuracy for Comorbid symptoms and mobile phone addiction. (A) Nonparametric bootstrapped difference test for strength. (B) Nonparametric bootstrapped difference test for bridge strength. Gray boxes indicate that node strength/bridge strength does not show significant differences, while black boxes indicate significant differences. The numbers within the white boxes (i.e., along the diagonal) represent the values of node strength/bridge strength. (C) Post-hoc analysis of the stability of node strength and bridge strength. The x-axis shows the percentage of the original sample used at each step, while the y-axis reflects the average correlations between the centrality metrics in the original network and those from the re-estimated networks after excluding progressively larger percentages of cases. The lines represent the correlations for strength and bridge strength. (D) Bootstrapped 95% confidence intervals for estimated edges. The red line indicates the edge estimated from the sample, and the gray area represents the 95% bootstrapped confidence interval. The x-axis shows the edges, with specific edges identified along the y-axis by the gray lines.


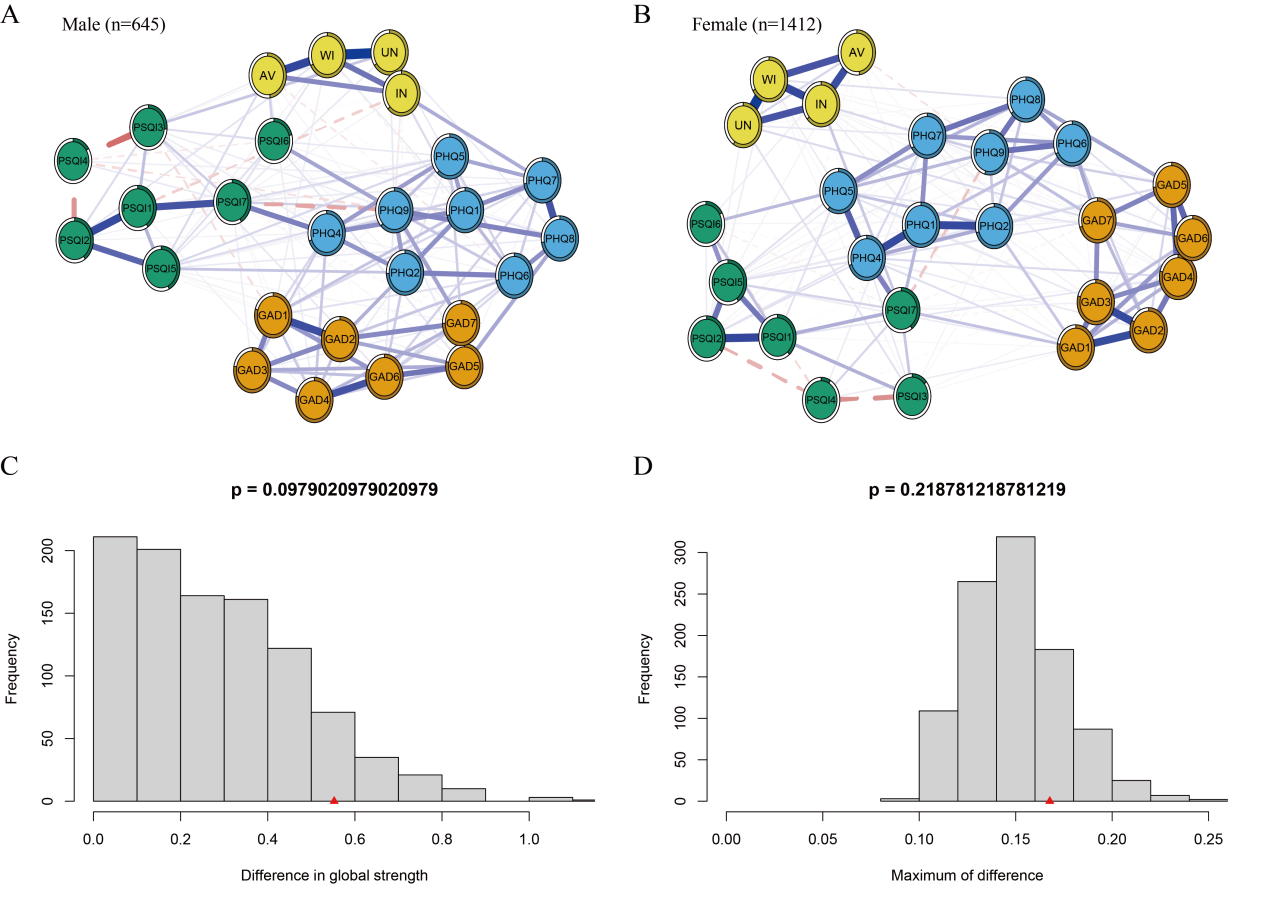


**Figure S6.** Network structure comparison based on gender. (A) Estimated network model for males (n = 645). (B) Estimated network model for females (n = 1412). (C) Bootstrap plot illustrating the difference in global strength, which was not statistically significant (global strength for males: 12.76; for females: 12.21; p = 0.102). (D) Bootstrap plot of the difference in network structure, showing no significant difference (M = 0.17, p = 0.199). The invariance of edge weights was assessed using permutation tests, yielding p values for each edge comparison. All Bonferroni-Holm adjusted p values were greater than 0.05.


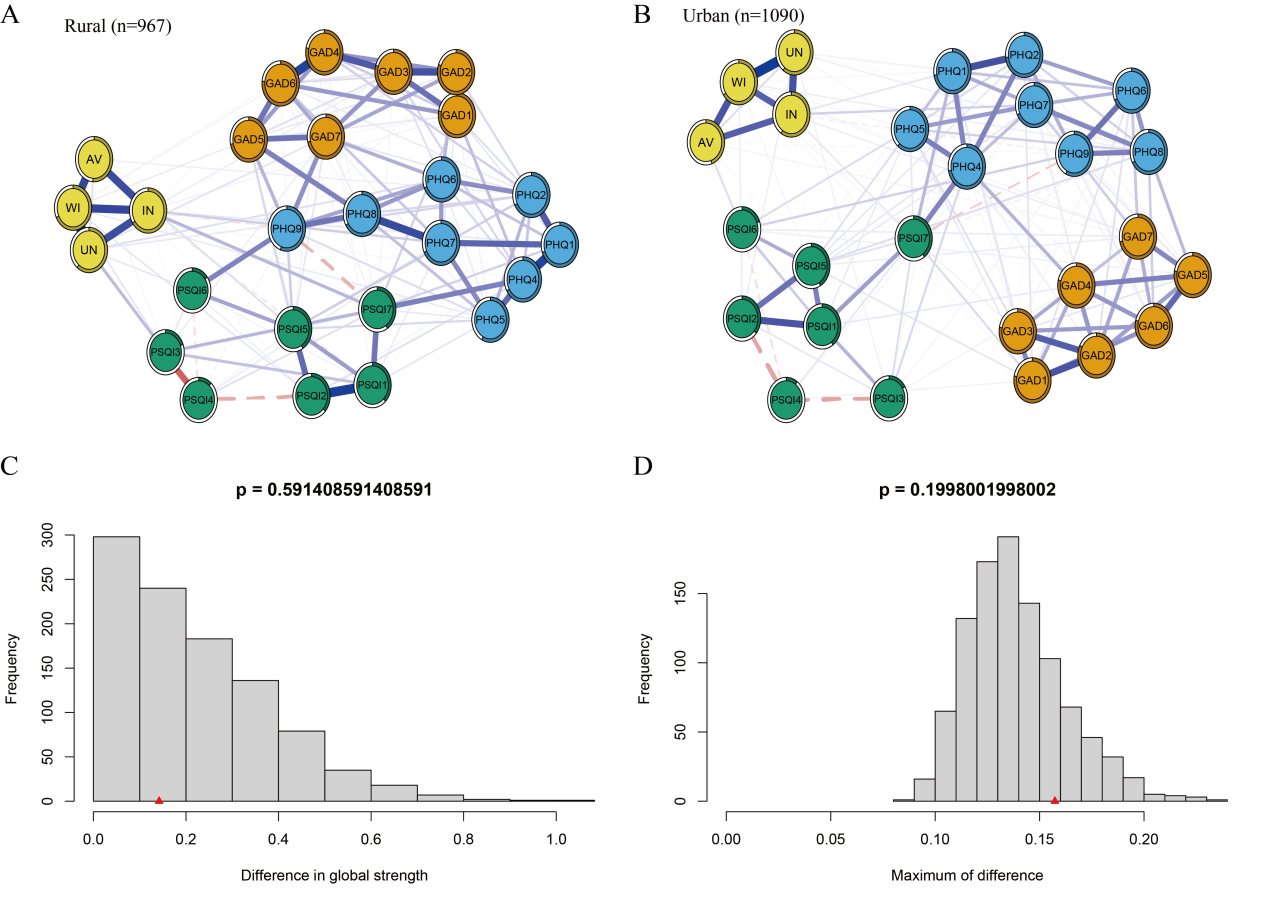


**Figure S7.** Network structure comparison based on residence. (A) Estimated network model for rural (n = 967). (B) Estimated network model for urban (n = 1090). (C) Bootstrap plot illustrating the difference in global strength, which was not statistically significant (global strength for rural: 12.12; for urban: 11.98; p = 0.590). (D) Bootstrap plot of the difference in network structure, showing no significant difference (M = 0.16, p = 0.220). The invariance of edge weights was assessed using permutation tests, yielding p values for each edge comparison. All Bonferroni-Holm adjusted p values were greater than 0.05.


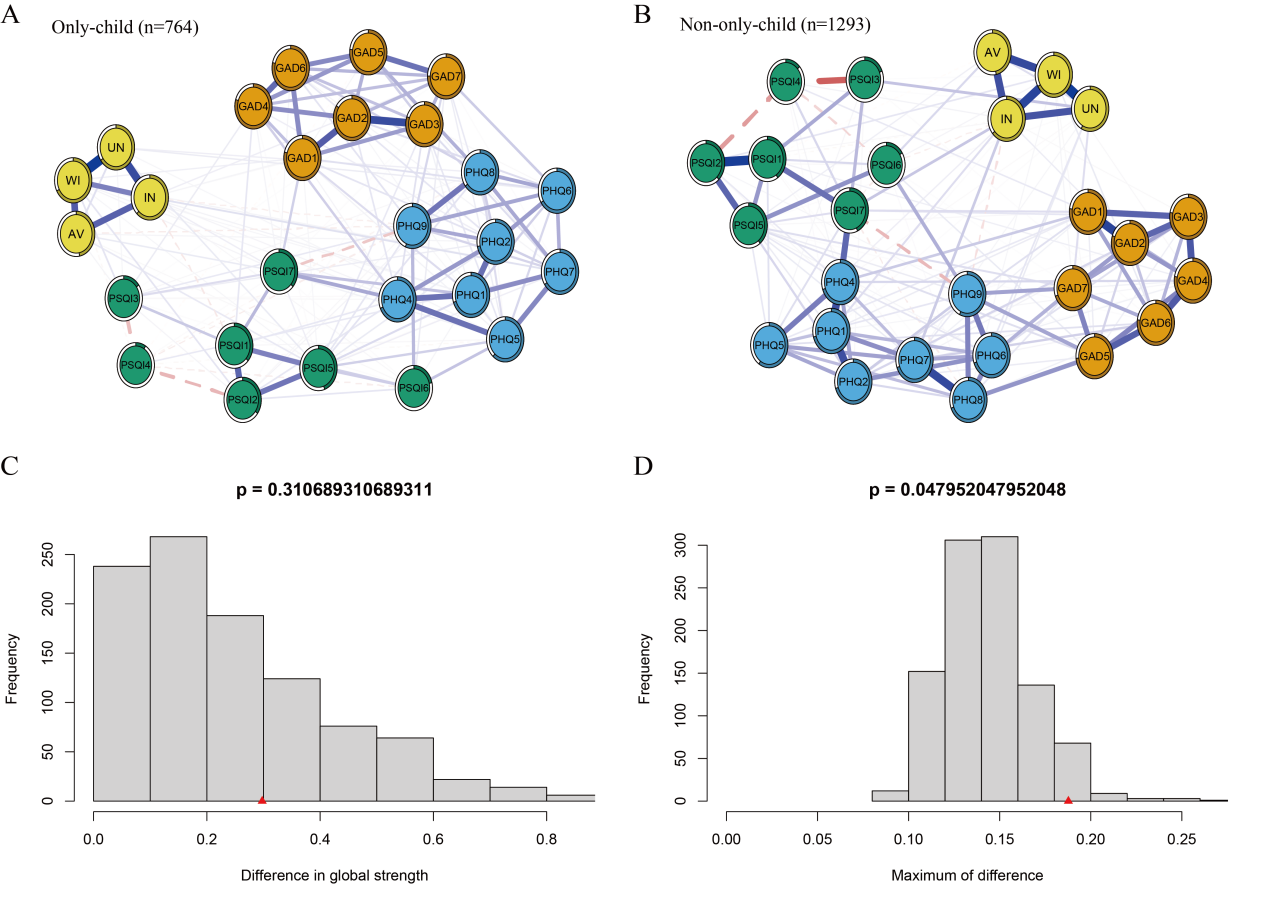
**Figure S8.** Network structure comparison based on family sibling status. (A) Estimated network model for Only Children (n = 764). (B) Estimated network model for Non-Only Children (n = 1293). (C) Bootstrap plot illustrating the difference in global strength, which was not statistically significant (global strength for Only Children: 11.95; for Non-Only Children: 12.24; p = 0.340). (D) Bootstrap plot of the difference in network structure, showing no significant difference (M = 0.19, p = 0.054). The invariance of edge weights was assessed using permutation tests, yielding p values for each edge comparison. All Bonferroni-Holm adjusted p values were greater than 0.05.

**Table S1.** Fundamental Information on Scales and Item Descriptive Statistics.

| **Items context** | **Mean** | **SD** | **Skewness** | **Kurtosis** | **Strength** | **Bridge strength** | **Predictability** |
| --- | --- | --- | --- | --- | --- | --- | --- |
| GAD1: Nervousness | 0.929 | 0.853 | 0.732 | -0.020 | 1.055 | 0.282 | 0.779 |
| GAD2: Uncontrollable worry | 0.818 | 0.869 | 0.910 | 0.144 | 1.132 | 0.134 | 0.815 |
| GAD3: Excessive worry | 0.901 | 0.879 | 0.774 | -0.096 | 1.093 | 0.174 | 0.787 |
| GAD4: Trouble relaxing | 0.849 | 0.886 | 0.852 | -0.033 | 1.048 | 0.141 | 0.783 |
| GAD5: Restlessness | 0.679 | 0.847 | 1.123 | 0.498 | 1.051 | 0.309 | 0.746 |
| GAD6: Irritability | 0.803 | 0.864 | 0.905 | 0.112 | 1.060 | 0.177 | 0.776 |
| GAD7: Feeling afraid | 0.706 | 0.858 | 1.114 | 0.518 | 0.950 | 0.318 | 0.710 |
| PHQ1: Anhedonia | 0.968 | 0.868 | 0.700 | -0.103 | 1.058 | 0.181 | 0.704 |
| PHQ2: Sad mood | 0.849 | 0.810 | 0.769 | 0.145 | 1.053 | 0.221 | 0.701 |
| PHQ4: Low energy | 1.033 | 0.895 | 0.602 | -0.359 | 1.120 | 0.425 | 0.690 |
| PHQ5: Abnormal appetite | 0.896 | 0.888 | 0.747 | -0.224 | 0.893 | 0.239 | 0.595 |
| PHQ6: Worthlessness | 0.759 | 0.874 | 0.980 | 0.158 | 1.005 | 0.241 | 0.684 |
| PHQ7: Poor concentration | 0.835 | 0.880 | 0.849 | -0.056 | 1.036 | 0.195 | 0.671 |
| PHQ8: Abnormal behavior and speech | 0.649 | 0.842 | 1.193 | 0.666 | 1.087 | 0.325 | 0.667 |
| PHQ9:Self-harm even suicide | 0.486 | 0.780 | 1.621 | 1.967 | 1.002 | 0.464 | 0.557 |
| PSQI1: Subjective sleep quality | 1.045 | 0.786 | 0.431 | -0.198 | 0.909 | 0.078 | 0.382 |
| PSQI2: Sleep latency | 1.362 | 0.938 | 0.174 | -0.853 | 0.831 | 0.110 | 0.360 |
| PSQI3: Sleep duration | 1.055 | 0.832 | 0.524 | -0.208 | 0.513 | 0.095 | 0.158 |
| PSQI4: Sleep efficiency | 2.114 | 0.919 | -0.779 | -0.305 | 0.544 | 0.084 | 0.103 |
| PSQI5: Sleep disturbance | 0.977 | 0.688 | 0.432 | 0.333 | 0.862 | 0.287 | 0.406 |
| PSQI6: Use of sleep medication | 0.187 | 0.589 | 3.437 | 11.483 | 0.472 | 0.201 | 0.163 |
| PSQI7: Daytime dysfunction | 1.641 | 1.013 | -0.207 | -1.053 | 1.033 | 0.656 | 0.395 |
| WI: Withdrawal | 10.220 | 3.954 | 0.136 | -0.615 |  |  |  |
| UN: Uncontrollability | 17.356 | 6.146 | 0.255 | -0.245 |  |  |  |
| IN: Inefficiency | 8.062 | 3.129 | 0.110 | -0.550 |  |  |  |
| AV: Avoidance | 8.077 | 3.062 | 0.213 | -0.487 |  |  |  |

**Table S2.** Correlation matrix of Anxiety, Depression and Sleep problems.

|  | **GAD1** | **GAD2** | **GAD3** | **GAD4** | **GAD5** | **GAD6** | **GAD7** | **PHQ1** | **PHQ2** | **PHQ4** | **PHQ5** | **PHQ6** | **PHQ7** | **PHQ8** | **PHQ9** | **PSQI1** | **PSQI2** | **PSQI3** | **PSQI4** | **PSQI5** | **PSQI6** | **PSQI7** |
| --- | --- | --- | --- | --- | --- | --- | --- | --- | --- | --- | --- | --- | --- | --- | --- | --- | --- | --- | --- | --- | --- | --- |
| **GAD1** | 0.000 |  |  |  |  |  |  |  |  |  |  |  |  |  |  |  |  |  |  |  |  |  |
| **GAD2** | 0.308 | 0.000 |  |  |  |  |  |  |  |  |  |  |  |  |  |  |  |  |  |  |  |  |
| **GAD3** | 0.196 | 0.267 | 0.000 |  |  |  |  |  |  |  |  |  |  |  |  |  |  |  |  |  |  |  |
| **GAD4** | 0.096 | 0.165 | 0.179 | 0.000 |  |  |  |  |  |  |  |  |  |  |  |  |  |  |  |  |  |  |
| **GAD5** | 0.014 | 0.089 | 0.046 | 0.173 | 0.000 |  |  |  |  |  |  |  |  |  |  |  |  |  |  |  |  |  |
| **GAD6** | 0.160 | 0.033 | 0.104 | 0.250 | 0.217 | 0.000 |  |  |  |  |  |  |  |  |  |  |  |  |  |  |  |  |
| **GAD7** | 0.000 | 0.136 | 0.128 | 0.045 | 0.203 | 0.120 | 0.000 |  |  |  |  |  |  |  |  |  |  |  |  |  |  |  |
| **PHQ1** | 0.033 | 0.000 | 0.016 | 0.000 | 0.000 | 0.000 | 0.000 | 0.000 |  |  |  |  |  |  |  |  |  |  |  |  |  |  |
| **PHQ2** | 0.045 | 0.036 | 0.000 | 0.021 | 0.011 | 0.030 | 0.000 | 0.265 | 0.000 |  |  |  |  |  |  |  |  |  |  |  |  |  |
| **PHQ4** | 0.051 | 0.013 | 0.018 | 0.065 | 0.000 | 0.000 | 0.000 | 0.262 | 0.145 | 0.000 |  |  |  |  |  |  |  |  |  |  |  |  |
| **PHQ5** | 0.029 | 0.000 | 0.026 | 0.000 | 0.000 | 0.007 | 0.008 | 0.072 | 0.079 | 0.211 | 0.000 |  |  |  |  |  |  |  |  |  |  |  |
| **PHQ6** | 0.012 | 0.064 | 0.039 | 0.000 | 0.000 | 0.000 | 0.103 | 0.096 | 0.160 | 0.015 | 0.019 | 0.000 |  |  |  |  |  |  |  |  |  |  |
| **PHQ7** | 0.010 | 0.021 | 0.000 | 0.000 | 0.008 | 0.051 | 0.000 | 0.182 | 0.011 | 0.062 | 0.162 | 0.136 | 0.000 |  |  |  |  |  |  |  |  |  |
| **PHQ8** | 0.000 | 0.000 | -0.035 | 0.000 | 0.150 | 0.054 | 0.027 | 0.000 | 0.068 | 0.000 | 0.086 | 0.164 | 0.248 | 0.000 |  |  |  |  |  |  |  |  |
| **PHQ9** | 0.000 | 0.000 | 0.000 | 0.000 | 0.060 | 0.006 | 0.112 | 0.000 | 0.103 | 0.000 | 0.024 | 0.173 | 0.040 | 0.197 | 0.000 |  |  |  |  |  |  |  |
| **PSQI1** | 0.000 | 0.000 | 0.000 | 0.031 | 0.000 | 0.000 | 0.000 | 0.000 | 0.000 | 0.000 | 0.037 | -0.010 | 0.000 | 0.000 | 0.000 | 0.000 |  |  |  |  |  |  |
| **PSQI2** | 0.000 | 0.000 | -0.008 | 0.000 | 0.005 | 0.000 | 0.000 | 0.018 | 0.000 | 0.044 | 0.000 | 0.000 | 0.023 | 0.011 | 0.000 | 0.319 | 0.000 |  |  |  |  |  |
| **PSQI3** | 0.000 | 0.000 | 0.000 | 0.012 | 0.000 | 0.010 | 0.000 | 0.000 | 0.025 | 0.032 | 0.000 | 0.014 | 0.000 | 0.000 | 0.002 | 0.112 | 0.011 | 0.000 |  |  |  |  |
| **PSQI4** | 0.023 | 0.000 | 0.000 | 0.000 | -0.005 | -0.011 | -0.004 | 0.000 | 0.000 | 0.000 | 0.000 | 0.000 | 0.035 | 0.000 | -0.007 | 0.000 | -0.143 | -0.199 | 0.000 |  |  |  |
| **PSQI5** | 0.012 | 0.000 | 0.000 | 0.000 | 0.031 | 0.000 | 0.045 | 0.016 | 0.054 | 0.001 | 0.050 | 0.000 | 0.026 | 0.016 | 0.036 | 0.170 | 0.225 | 0.000 | 0.000 | 0.000 |  |  |
| **PSQI6** | 0.000 | 0.000 | 0.000 | 0.000 | 0.015 | 0.000 | 0.020 | 0.000 | 0.000 | -0.007 | 0.041 | 0.000 | 0.000 | 0.000 | 0.118 | 0.059 | 0.021 | 0.000 | -0.061 | 0.130 | 0.000 |  |
| **PSQI7** | 0.067 | 0.000 | 0.032 | 0.013 | -0.025 | 0.008 | 0.000 | 0.098 | 0.000 | 0.193 | 0.041 | 0.000 | 0.021 | -0.033 | -0.125 | 0.172 | 0.002 | 0.096 | 0.057 | 0.050 | 0.000 | 0.000 |

**Table S3.** Bridge Strength linking mobile phone addiction to anxiety, depression, and sleep Problems.

|  | **Anxiety community** | **Depression community** | **Sleep problems community** |
| --- | --- | --- | --- |
| **Withdrawal** | 0.074 | 0.101 | 0.082 |
| **Uncontrollability** | 0.076 | 0.117 | 0.195 |
| **Inefficiency** | 0.114 | 0.284 | 0.216 |
| **Avoidance** | 0.009 | 0.000 | 0.079 |
